# Delayed Arterial Hemorrhage From a Lumbar Artery Following Inferior Vena Cava Filter Placement: A Case Report

**DOI:** 10.7759/cureus.60668

**Published:** 2024-05-20

**Authors:** Audrey Bui, Ross Gillan, Austin Vaughn, Arden Bui, Douglass Sherard

**Affiliations:** 1 Osteopathic Medicine, Lake Erie College of Osteopathic Medicine, Bradenton, USA; 2 Interventional Radiology, Ascension St. Vincent's Hospital, Jacksonville, USA

**Keywords:** ivc filter complications, interventional radiology knowledge, lumbar artery hemorrhage, ivc filter complication, radiology interventional

## Abstract

Pulmonary embolism (PE) is a feared complication of deep venous thrombosis (DVT) that can lead to respiratory distress and even death. The mainstay of preventing PE is anticoagulation, but other strategies exist. Inferior vena cava (IVC) filters are an alternative strategy for PE prophylaxis in individuals who may have contraindications to receiving anticoagulation. Although the placement of an IVC filter is a minimally invasive and typically uncomplicated procedure, all procedures have their risks. We present a case of a 35-year-old woman who experienced a rare complication of IVC filter placement and suffered a retroperitoneal hemorrhage. The patient underwent placement of an IVC filter for PE prophylaxis before a scheduled sleeve gastrectomy. Hours after placement, she returned with new symptoms and signs of blood loss. She was found to have a retroperitoneal hematoma due to bleeding from a lumbar artery that was penetrated by a strut of the filter. Arterial hemorrhage from a lumbar artery is a rare complication of IVC filter placement, and it can result in poor outcomes for the patient. We aim to increase awareness of this rare but dangerous complication to improve recognition and patient outcomes in cases of delayed arterial hemorrhage following IVC filter placement.

## Introduction

Venous thromboembolism (VTE) is a common disease with high morbidity and mortality. It includes both deep vein thrombosis (DVT) and pulmonary embolism (PE), with PE being the primary cause of mortality. Inferior vena cava (IVC) filter placement is an alternative prophylaxis against PE in patients in which the use of anticoagulation may be contraindicated or ineffective. It is a relatively low-risk procedure that has seen an upward trend in procedures performed each year [1]. Complications of IVC filter placement are procedure-related or post-procedural. Common procedure-related complications include access site bleeding and access site thrombosis [2,3]. Post-procedural complications can include filter migration, thrombosis, and IVC perforation [4-6]. An extremely rare but dangerous complication following IVC filter insertion is delayed arterial hemorrhage, which has been described in five previous case reports [7-11]. We report a case of retroperitoneal arterial hemorrhage with hematoma formation due to penetration of a lumbar artery following IVC filter placement. With the exponential rise in the use of IVC filters, there is an increased value in exploring and reporting potential complications, especially those with poor patient outcomes.

## Case presentation

**Figure 1:**
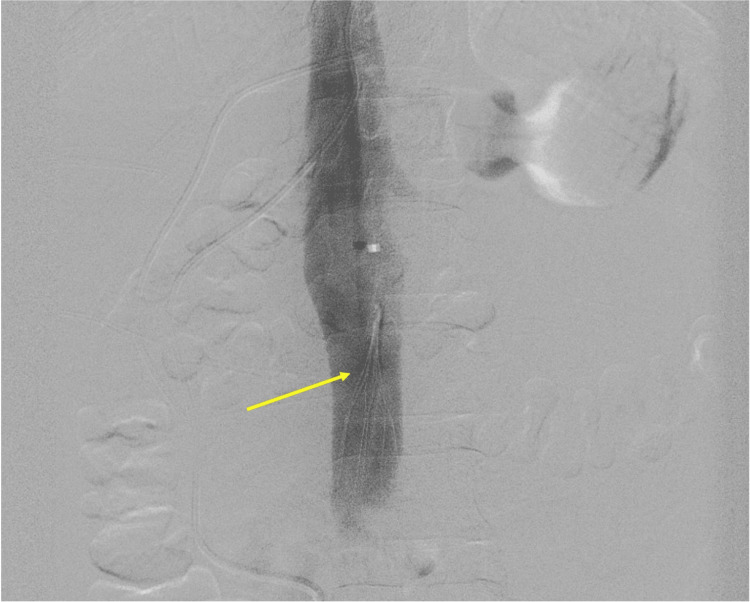
Intra-operative angiogram demonstrating IVC filter (yellow arrow) placement IVC - inferior vena cava

A 35-year-old morbidly obese female with a history of pulmonary embolism was preparing for a sleeve gastrectomy. Due to her history of PE, she was taking warfarin, which was to be discontinued and replaced perioperatively with an IVC filter to ensure a safe operation. She presented to interventional radiology (IR) for routine IVC filter placement. Her preoperative hemoglobin was 11.0 g/dL, INR was 1.5, and her vitals were stable. Postoperative imaging revealed proper placement (Figure 1). She was discharged an hour after placement with no sign of complication. 

**Figure 2:**
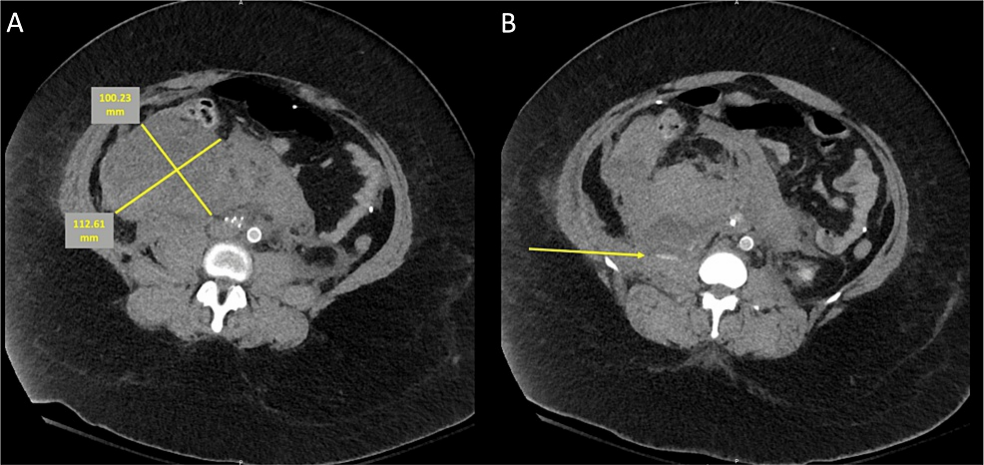
CT post-IVC filter placement demonstrating hematoma formation (A) and extravasation of blood from the lumbar branch (B) IVC - inferior vena cava

**Figure 3:**
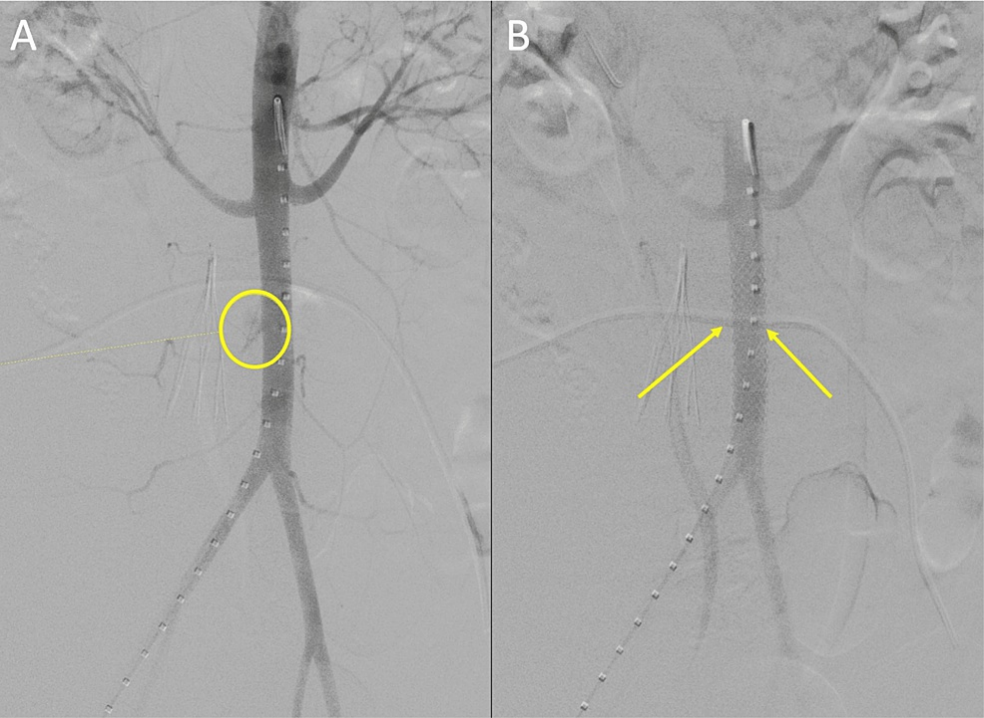
Intra-operative aortogram post-IVC filter placement demonstrating extravasation of blood pre-stent placement (A) and reestablished flow post-stent placement (B) IVC - inferior vena cava

Hours later, the patient returned with sudden onset right-sided flank pain. She was hemodynamically stable, but her hemoglobin was found to be severely low at 6.0 g/dL. A CT scan of the abdomen and pelvis at that time showed a large hematoma in the retroperitoneal cavity (Figure 2A), and a CT angiography of the abdomen demonstrated extravasation from a lumbar arterial branch (Figure 2B). It was determined that a filter strut had penetrated through the caval wall and injured a nearby lumbar artery. The patient became hypotensive and was taken emergently to the operating room for endovascular stent placement. In the operating room, the right common femoral artery was used for access, and a balloon expandable stent was deployed in the mid-aorta. After stent deployment, subsequent imaging demonstrated no extravasation of contrast medium (Figure 3A; Figure 3B). The procedure was complicated by acute thrombosis of the right common femoral artery and dissection of the distal external iliac artery. The femoral artery was treated with patch angioplasty and thrombectomy, and the external iliac dissection was treated with a stent. The patient was taken to the ICU for further resuscitation and eventually recovered well. 

## Discussion

This case demonstrates a rare complication of IVC filter placement. Although caval penetration appears to be one of the more common complications of IVC filters, a lumbar artery is rarely involved. A systematic literature review by Jia et al. found an occurrence of caval wall penetration in 19% of IVC filter patients but believed the true incidence of caval penetration to be higher [12]. In the same study, investigators reported that of the 1699 cases reporting caval penetration, there were only 305 instances of involvement of adjacent structures, with the lumbar artery only being affected in six of these cases [12]. Adjacent structures that have been involved in caval penetrations have more often included the duodenum, lumbar vertebrae, and aorta [12]. Since this complication is so infrequently reported, a physician’s clinical suspicion of caval penetration and involvement of a lumbar artery may be very low and could delay appropriate treatment when it occurs.

The incidence of different complications, such as caval penetration and injury to adjacent structures, varies between different types of filters. There are many different designs of IVC filters, and the type of filter may determine whether or not the device is retrievable and/or if retrieval can be performed safely. In an observational study by Wang et al., rates of IVC perforation were higher in retrievable devices compared to permanent, and retroperitoneal structures were more commonly involved with conical retrievable devices [13]. The length of the indwelling of the catheter also affects the risk of IVC penetration. Lee et al. demonstrated a 15-fold increased risk of penetration when indwelling is greater than 20 days [14].

Most cases of caval penetration are asymptomatic and may even be found incidentally on abdominal CT or other intra-abdominal surgeries [15]. Although symptomatic cases of caval penetration are uncommon and the involvement of a lumbar artery is even more rare, the danger of this complication warrants further research on the topic. Providers should have a high level of suspicion for delayed arterial hemorrhage from a lumbar artery when a patient status post IVC filter placement presents with signs of a retroperitoneal bleed, such as new back or flank pain.

## Conclusions

Deployment of an IVC filter is a strategy to prevent PE in patients who may have contraindications to anticoagulation therapy. Although there is a relatively low complication rate, with the rise in the use of IVC filters, there is an increased value in exploring and reporting potential complications. We present a case of lumbar artery penetration by an IVC filter hours after its placement. Although this patient was treated in a timely manner and made a full recovery, any delay in diagnosis and treatment could have led to more serious or long-term consequences. Despite being a very rare complication, its potential consequences warrant a high index of suspicion when a patient status post IVC filter placement presents with new symptoms and signs of bleeding. Additionally, the possibility of penetration of a nearby artery should be considered in future design of IVC filters.
